# COVID-19 vaccine utilisation among people living with HIV on antiretroviral therapy in Zimbabwe

**DOI:** 10.4102/jphia.v16i1.664

**Published:** 2025-03-11

**Authors:** Talent Tapera, Clifford Odimegwu, Tatenda Makoni, Waraidzo Mukuwapasi, Vivian Chitiyo, Gilton Kadziyanike, Abigail Mutsinze, Nicola Willis, Garikayi Chemhaka, Million Phiri

**Affiliations:** 1 Demography and Population Studies Programme, Schools of Public Health and Social Sciences, University of the Witwatersrand, Johannesburg, South Africa; 2 Zimbabwe National Network of People Living with HIV (ZNNP+), Harare, Zimbabwe; 3 Zvandiri, Harare, Zimbabwe; 4 Department of Statistics and Demography, Faculty of Social Sciences, University of Eswatini, Mbabane, Eswatini; 5 Department of Population Studies, Faculty of Humanities, University of Zambia, Lusaka, Zambia

**Keywords:** HIV, COVID-19, COVID-19 vaccination, antiretroviral therapy, Community Adolescent Treatment Supporters

## Abstract

**Background:**

Several studies have now highlighted COVID-19 vaccination hesitancy in the public. However, not much is known about COVID-19 vaccination amoung people living with HIV (PLHIV).

**Aim:**

This study aimed to examine the predisposition of PLHIV on antiretroviral therapy to utilise the COVID-19 vaccine.

**Setting:**

The study was done in urban and rural communities of Zimbabwe

**Methods:**

The study utilised a concurrent triangulation design of which only one data collection phase was used. The quantitative data were collected from 2157 PLHIV on antiretroviral viral therapy through a structured interviewer-administered questionnaire. On the other hand, qualitative data were collected through in-depth interviews.

**Results:**

The study found a higher proportion of COVID-19 vaccine utilisation among adults in comparison to adolescents and young people. Fear of side effects contributed to the most common reason for not getting vaccinated. Adolescents aged 15–19 years were significantly less likely to receive the COVID-19 vaccine (adjusted odds ratios [aOR] = 0.58; 95% CI: 0.41–0.83). Being a previous contact of a COVID-19 case was significantly associated with higher odds of COVID-19 vaccination (aOR = 3.43; 95% CI: 1.92–6.10). Additionally, living in a rural area was associated with higher odds of COVID-19 vaccination among PLHIV (aOR = 1.38; 95% CI: 1.05–1.83).

**Conclusion:**

There is need to broaden to speak to the role of families, communities and healthcare workers ensuring adolescent and youth-friendly information and support for vaccinations.

**Contribution:**

The COVID-19 vaccination rates were lower among adolescents and young people compared to adults living with HIV. Adolescents and young people face challenges in accessing adolescent-friendly health services.

## Introduction

Recent studies have documented the heightened risk among people living with HIV (PLHIV) after contracting COVID-19. A retrospective study in the United Kingdom found that PLHIV had a higher risk of COVID-19 death than those without an HIV, even after adjusting for age and sex.^1^ Similarly, in Africa, a systemic review and meta-analysis showed an increased risk of mortality among PLHIV who also had acute respiratory tract infections.^2^ These findings were also consistent with a study in South Africa involving public sector patients, which showed that increased COVID-19 mortality was associated with HIV, previous and current tuberculosis, older age, male sex, diabetes, hypertension and chronic kidney disease.^3^

In the fight against the HIV epidemic, there is need to ensure that all PLHIV access treatment services to guarantee holistic reduction of HIV morbidity and mortality, as well as a reduction in new infections through suppressed viral loads, which make HIV untransmissible. Potentially, the fight against the HIV epidemic may be more difficult if there is no full protection against emerging epidemics such as COVID-19. Thus, this study examined the predisposition of PLHIV on antiretroviral therapy (ART) to utilise the COVID-19 vaccine and factors that influence their decision in Zimbabwe. The study also highlights if there are differences in COVID-19 vaccine coverages and predictors between adolescents and young people (15–24) and adults (25+).

Zimbabwe has a generalised HIV epidemic and is home to 1.35 million PLHIV, including 1.27 million adults and 76 300 children. An estimated 1.35 million people were living with HIV in 2019, with 5.6% being children 0–14 years. Among adults 15+ years living with HIV, 60% were females. Zimbabwe since the outbreak of COVID-19 has recorded 264 127 confirmed COVID-19 cases and 5668 deaths in the second week of March 2023.

## Methods and data

### Data source and study design

A mixed-method approach utilising a concurrent triangulation design was used. A concurrent study design is an approach in which both quantitative and qualitative data are collected at the same time. The quantitative data were collected from people who are living with HIV on ART through a structured questionnaire. The quantitative questionnaire asked about all the predisposing, enabling, need and environmental factors as they relate to accessing or not accessing the expected HIV treatment services per each sample person living with HIV on ART. On the qualitative data collection modality, qualitative data were collected through in-depth interviews with purposively sampled key stakeholders such as community HIV volunteers, district HIV focal persons and district health promotion officers. The analysis of the quantitative and the qualitative data was conducted separately yet concurrently. The findings were integrated during the results and interpretation phases of the study.

The quantitative questionnaire asked about all the predisposing, enabling, need and environmental factors as they relate to getting vaccinated for COVID-19 from the year 2021 to December 2022.^4^ The questionnaire was administered to adolescents and young people (15–24) by Community Adolescent Treatment Supporters (CATS) from Zvandiri, while data for adults were administered by Community Health Agents (CHAs) from Zimbabwe National Network of People Living with HIV (ZNNP+). The total sample size from both the CATS and CHAs surveys was 2157, of which 791 were adolescents and young people (15–24) and 1366 were adults (25+). On the qualitative data collection modality, qualitative data collection was collected through in-depth interviews with purposively sampled key stakeholders such as 24 HIV volunteers, 6 district HIV focal persons and 6 health promotion officers. The analysis of the quantitative and the qualitative data was conducted separately but yet concurrently.^4^

### Study variables

#### Outcome variables

The dependent variable for this study was the COVID-19 vaccine utilisation among PLHIV. Each of the outcome variables was coded as binary, with ‘1’ representing utilisation of the COVID-19 vaccine and ‘0’ representing not yet vaccinated for COVID-19.^4^

#### Independent variables

**FIGURE 1:**
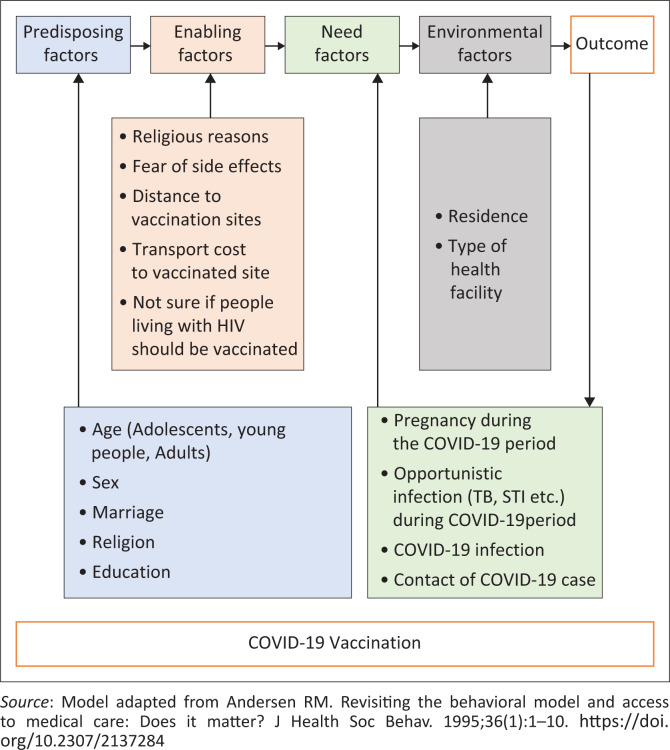
The health utilisation model for COVID-19 vaccination among people living with HIV who are on ART in Zimbabwe.

The study was premised on the fifth version of the Anderson healthcare services utilisation model, which argues that the actual use of healthcare services is a function of three factors: predisposing, enabling and need factors. Firstly, the predisposing factors include sociocultural characteristics of individuals that exist prior to their illness. These characteristics include demographic factors such as age and gender, as well as social structural variables such as education, among others. Secondly, the enabling factors are those that involve the logistical aspects of obtaining care such as the resources available, whether individually or in a community.^4^ These may include personal or family resources, income, health insurance, access to a regular source of care, travel, extent and quality of social relationships. At community level, these may imply available health personnel and facilities and waiting time among others. Thirdly, the need factors are the most immediate causes of health service use, derived from functional and health problems that generate the need for healthcare services.^5^ Figure 1 shows how the Anderson Model was adopted to explain the predictors of COVID-19 vaccination among PLHIV who are on ART.^5^

The dependent variable was defined as receiving COVID-19 vaccination (yes, no). The predisposing factors included age (15–19, 20–24, 25–39, 40–54, 55+), sex (male and female), marital status (not married, married), religion (Roman Catholic, Apostolic sect, Pentecostal and Protestant, other religions) and education (primary, secondary, tertiary, do not know and did not attend school and no answer). The enabling factors included religious reasons, fear of side effects, distance and transport cost to vaccination sites, and being not sure if PLHIV should be vaccinated. The need factors included pregnancy during COVID-19 study period for females, opportunistic infection during COVID-19 period, COVID-19 infection and contact with a COVID-19 case, while environmental factors included residence (urban, rural) and type of health facility (central and provincial health facility, district health facility, primary healthcare facility).

### Statistical analysis

Statistical analysis was performed using Stata version 17 software with 5% level of significance. Frequency distributions and Chi-square test of association were used at the univariate and bivariate levels of analysis. Multivariate binary logistic regression was used to identify the predictors of utilising the COVID-19 vaccine among PLHIV. All quantitative analyses were conducted at α = 0.05. Adjusted odds ratios (aOR) with corresponding *p*-values were reported. All covariates from the bivariate analysis were included in the multivariate analyses regardless of their significance. Thematic analysis was used to analyse the qualitative data to identify the factors responsible for utilisation of HIV care and treatment services in Zimbabwe. Qualitative analysis was conducted using Nvivo version 12.

### Ethical considerations

Ethical clearance to conduct this study was obtained from the Medical Research Council of Zimbabwe under reference number MRCZ/A/2868 and also by the University of the Witwatersrand Ethics Committee (Medical) under reference number M220425. Assurance of anonymity and confidentiality of their results was guaranteed to the survey participants. Furthermore, participation in the data collection process was voluntary.

## Results

### Sociodemographic characteristics of study participants

A total of 2157 PLHIV aged 15 years and older, who were on ART before the advent of COVID-19, were included in the study, as reported in Table 1. The majority of participants were adults aged 25 years and above (63.3%), while adolescents and young people made up 36.7%. Most participants were female (62.6%), with 37.4% being male. A significant proportion, 978 participants (45.3%), were single, 42.2% were married and 12.4% were widowed. More than half of the study participants (63.7%) were from rural areas and 36.3% were from urban areas. Pentecostal and Protestant represented the majority at 34.96%. Majority (66.1%) had gone through secondary education. Most participants (69.9%) accessed their HIV services from primary healthcare facilities. Additionally, the majority of participants had been vaccinated for COVID-19 (85.8%).

**TABLE 1:** Distribution of sociodemographic characteristics of study participants (*N* = 2157).

Categories	Frequency	%
**Age (years)**		
15–19	326	15.11
20–24	465	21.56
25–39	513	23.78
40–54	604	28.00
55+	249	11.54
**Sex**		
Male	806	37.37
Female	1351	62.63
**Marital status**		
Not married	1246	57.77
Married	911	42.23
**Residence**		
Rural	1374	63.70
Urban	783	36.30
**Religion**		
Roman Catholic	225	10.43
Apostolic sect	475	22.02
Pentecostal and Protestant	754	34.96
Other religions	703	32.96
**Education**		
Primary	492	22.81
Secondary	1426	66.11
Tertiary	129	5.98
Do not know/Did not attend school/No answer	110	5.10
**Type of health facility where HIV services are being accessed**		
Central/Provincial health facility	94	4.30
District health facility	556	25.70
Primary healthcare facility	1507	69.87
**Vaccinated for COVID-19**		
Yes	1851	85.81
No	306	14.19

### Utilisation of the COVID-19 vaccine among people living

Eighty-six per cent of the study participants living with HIV had been vaccinated for COVID-19. Table 2 shows proportion of utilisation of the COVID-19 vaccine among PLHIV by predisposing, need and environmental factors.

**TABLE 2:** Proportion of people living with HIV vaccinated for COVID-19 by predisposing, need and environmental factors (*N* = 2157).

Categories	Total	Vaccinated	Proportion (%)	Chi^2^	*p*-value
**Predisposing factors**					
**Age (years)**	-	***	-	121.59	0.00
15–19	326	227	70	-	-
20–24	465	375	89	-	-
25–39	513	454	89	-	-
40–54	604	563	93	-	-
55+	249	232	93	-	-
**Sex**	-	-	-	2.61	0.11
Male	806	679	84	-	-
Female	1351	1172	86	-	-
**Marital status**	-	***	-	21.64	0.00
Not married	1246	1032	82	-	-
Married	911	819	90	-	-
**Religion**	-	-	-	5.17	0.16
Roman Catholic	225	188	83	-	-
Apostolic sect	475	421	89	-	-
Pentecostal and Protestant	754	637	84	-	-
Other religions	703	605	86	-	-
**Education**	-	-	-	2.79	0.42
Primary	492	431	88	-	-
Secondary	1426	1213	85	-	-
Tertiary	129	144	88	-	-
Do not know/Did not attend school/No answer	110	93	85	-	-
**Need factors**					
**Pregnancy in the past 24 months**	-	-	-	0.21	0.64
Yes	196	168	86	-	-
No	1155	1004	87	-	-
**Opportunistic infection in the past 24 months**	-	**	-	10.91	0.001
Yes	247	229	93	-	-
No	1910	1622	85	-	-
**COVID-19 infection in the past 24 months**	-	***	-	14.36	0.00
Yes	315	292	93	-	-
No	1842	1559	85	-	-
**Contact of a COVID-19 case in the past 24 months**	-	***	-	39.38	0.00
Yes	417	398	95	-	-
No	1740	1453	84	-	-
**Environmental factors**					
**Residence**	-	***	-	13.78	0.00
Rural	1374	1208	88	-	-
Urban	783	643	82	-	-
**Type of health facility where HIV services are being accessed**	-	***	-	20.18	0.00
Central/Provincial health facility	94	70	74	-	-
District health facility	556	502	90	-	-
Primary healthcare facility	1507	1279	84	-	-

*** , *p* < 0.001;

** , *p* < 0.01

### Predisposing factors

Age (*x*^2^ = 121.59; *p* = 0.00) and marital status (*x*^2^ = 21.64; *p* = 0.00) were the predisposing factors that were significantly associated with getting vaccinated for COVID-19 among PLHIV using bivariate analysis. We found that the adolescent age group (15–19) had the least proportion (70%) of PLHIV vaccinated for COVID-19. More so, the young people (20–24) was the second ranked (81%), whereas older age groups had vaccination rates above 89%. In terms of sex, males had a lower proportion of vaccination (84%) compared to females (84%). People living with HIV who were not married least proportion of vaccination of 82% compared to 90% among the married. The Roman Catholic and Pentecostal and Protestant had the least proportion of vaccination with 83% and 84%, respectively. People living with HIV who had attained secondary or tertiary education had a lower vaccination rate (85%) compared to those with primary education (88%).

### Need factors

Opportunistic infection in the past 24 months (*x*^2^ = 10.91; *p* = 0.001), COVID-19 infection in the past 24 months (*x*^2^ = 14.36; *p* = 0.00) and contact with a COVID-19 case in the past 24 months (*x*^2^ = 39.38; *p* = 0.00) were the need factors that were significantly associated with getting vaccinated for COVID-19 among PLHIV using bivariate analysis. Pregnancy showed a marginal difference in vaccine rates, with 86% of pregnant females and 87% of non-pregnant females vaccinated. People living with HIV who had not had an opportunistic infection in the past 24 months had a lower proportion of 85% compared to 93% of those who had. A similar picture was seen among PLHIV who had not had a COVID-19 infection in the past 24 months having a lower utilisation proportion of 85% compared to 93% of those who had. People living with HIV who had not been in contact with a COVID-19 case in the past 24 months had a lower utilisation proportion of 84% compared to 95% of those who had been in contact.

In-depth interviews confirmed that in terms of pregnancy. ‘It was difficult for those who were pregnant living with HIV as they were not sure if it is safe to take while pregnant’ (community volunteer in-depth interview).

### Environmental factors

Residence (*x*^2^ = 13.78; *p* = 0.00) and type of health facility (*x*^2^ = 20.18; *p* = 0.00) were the environmental factors that were significantly associated with getting vaccinated for COVID-19 among PLHIV using bivariate analysis. The proportion of COVID-19 vaccine utilisation among PLHIV was higher in rural areas (88%) compared to 82% in rural areas. Moreover, PLHIV accessing their health services at central and provincial hospitals had a lower vaccine utilisation proportion (74%) in comparison to those accessing at district health facilities (90%) and primary healthcare facilities (84%).

### Enabling factors

Fear of side effects contributed to be the most common (48%) reason for not getting vaccinated among the study participants. This was followed by religious reasons (22%) and uncertainty about whether PLHIV should get vaccinated (15%). Distance to vaccination sites and transport costs were the least mentioned reasons, with less than 2% of participants citing them, while 13% provided other reasons.

The aforementioned reasons in the quantitative findings were also explained in the qualitative in-depth interviews, where the fear of side effects was confirmed by community volunteers, with 90% agreeing that this was a significant concern:

‘We struggled with issues of false information that vaccines were not safe for people living with HIV, hence there was hesitance to get vaccinated.’ (Community volunteer, In-depth interview Participant CV002, Male)

As such, vaccination among PLHIV then started to increase following vaccination campaigns where this fear was properly addressed. Religious reasons were also found to have influenced some hesitancy in vaccination among PLHIV:

‘Information that about the vaccine being associated with Satanism made it difficult for some to get the COVID-19 vaccine.’ (Community volunteer, In-depth interview Participant CV004, Female)

### Multivariate analysis of utilisation of the COVID-19 vaccines among people living with HIV

Regarding predisposing factors, adolescents aged 15–19 were significantly associated with less likelihoods of getting COVID-19 vaccination (aOR = 0.58; 95% CI: 0.41–0.83) in comparison with their 20–24 counterparts. Older age groups, aged 40–45 years (aOR = 3.29; 95% CI: 1.94–5.57) and 55+ years (aOR = 3.29; 95% CI: 1.94–5.57), were associated with higher odds of COVID-19 vaccination among PLHIV in comparison with the 20–24 age group. Regarding need factors, being a previous contact of a COVID-19 case was significantly associated with higher odds of COVID-19 vaccination (aOR = 3.43; 95% CI: 1.92–6.10) in comparison with not getting vaccinated. Regarding environmental factors, living in a rural area was associated with higher odds of COVID-19 vaccination among PLHIV (aOR = 1.38; 95% CI: 1.05–1.83) in comparison with their urban counterparts.

### Comparison of utilisation of COVID-19 vaccination among people living with HIV by age

Among PLHIV who vaccinated (*n* = 1851), the study found a higher proportion of utilisation of COVID-19 vaccine among adults 25+ years, which was 91% (1249/1366), in comparison to adolescents and young people, which was 76% (602/791). Table 3 shows the differences in COVID-19 vaccine utilisation by predisposing, need and environmental factors. Additionally, in-depth interviews showed that the severity of COVID-19 in older age groups led to COVID-19 vaccine messaging being more targeted towards these age groups:

‘COVID-19 had more severe consequences in older age bands hence messaging followed suit for the protection of those who were mostly vulnerable …’ (Community volunteer, Participant CV005, Male)

**TABLE 3:** Differences in vaccine utilisation by age among people living with HIV.

Categories	Adolescents and young people (15–24 years) living with HIV (*N* = 791)			Adults (25+ years) living with HIV (*N* = 1366)			Chi^2^	*p*-value
	Total	Utilisation		Total	Utilisationy			
		*n*	%		*n*	%		
**Predisposing factors**								
**Sex**	-	-	-	-	-	-	42.29	0.00***
Male	378	284	75	428	395	92	-	-
Female	413	318	77	938	854	91	-	-
**Marital status**	-	-	-	-	-	-	247.12	0.00***
Not married	658	493	75	588	539	92	-	-
Married	133	109	82	778	710	91	-	-
**Religion**	-	-	-	-	-	-	73.77	0.00***
Roman Catholic	78	61	78	147	127	86	-	-
Apostolic sect	123	97	79	352	324	92	-	-
Pentecostal and Protestant	243	169	70	511	468	92	-	-
Other religions	347	275	79	356	330	93	-	-
**Education**	-	-	-	-	-	-	197.46	0.00***
Primary	65	34	52	427	397	93	-	-
Secondary	579	445	77	847	768	91	-	-
Tertiary	83	70	84	46	44	96	-	-
Do not know/Did not attend/No answer	64	53	83	46	40	87	-	-
**Need factors**								
**Pregnancy in the past 24 months**	-	-	-	-	-	-	0.01	0.94
Yes	59	46	78	137	122	89	-	-
No	354	272	77	801	732	91	-	-
**Opportunistic infection in the past 24 months**	-	-	-	-	-	-	46.96	0.00***
Yes	35	29	83	212	200	94	-	-
No	756	573	76	1154	1049	91	-	-
**COVID-19 infection in the past 24 months**	-	-	-	-	-	-	21.38	0.00***
Yes	72	61	85	243	231	95	-	-
No	719	541	75	1123	1018	91	-	-
**Contact of a COVID-19 case in the past 24 months**	-	-	-	-	-	-	25.04	0.00***
Yes	97	88	91	320	310	97	-	-
No	694	514	74	1046	939	90	-	-
**Environmental factors**								
**Residence**	-	-	-	-	-	-	48.56	0.00***
Rural	443	326	74	931	882	95	-	-
Urban	348	276	79	435	367	84	-	-
**Type of health facility where HIV services are being accessed**	-	-	-	-	-	-	193.61	0.00***
Central/provincial health facility	78	59	76	16	11	69	-	-
District health facility	80	62	78	476	440	92	-	-
Primary healthcare facility	633	481	76	874	798	91	-	-

*** , *p* < 0.001

Regarding predisposing factors, there were significant differences in sex (*x*^2^ = 42.29, *p* = 0.00), marital status (*x*^2^ = 247.12, *p* = 0.00), religion (*x*^2^ = 73.77, *p* = 0.00) and education (*x*^2^ = 197.46, *p* = 0.00) between adolescents and young people (15–24) and adults (25+) living with HIV as it relates to COVID-19 vaccination. Both adolescents and young people (15–24) and adults (25+) had males with a lower proportion of non-utilisation compared to females. Among adolescents and young people, vaccine utilisation was 75% and 77% among males and females, respectively, whereas among adults, it was 92% and 91% among males and females, respectively. Both adolescents and young people (75% compared to 82%) and adults (92% compared to 91%) who had not married with had lower vaccine utilisation, with a slight difference observed in adults. Regarding religion, the Pentecostal and Protestant group had lower vaccine utilisation among adolescents and young people (70%) as compared to adults (92%). Among adults, the lowest utilisation was among the Roman Catholic (86%). Adolescents and young people who had attained only primary education had a lower proportion of vaccine utilisation (52%) compared to adults with primary education (93%).

Regarding need factors, there were significant differences in opportunistic infection in the past 24 months (*x*^2^ = 46.96, *p* = 0.00), COVID-19 infection in the past 24 months (*x*^2^ = 21.38, *p* = 0.00), and contact with a COVID-19 case in the past 24 months (*x*^2^ = 25.04, *p* = 0.00) between adolescents and young people (15–24) and adults (25+) living with HIV as it relates to COVID-19 vaccination. Among participants who had been pregnant during COVID-19 restrictions period, 78% vaccinated for COVID-19 among adolescents and young people in comparison to 89% among adults. Among adolescents and young people who had an opportunistic infection, utilisation of the COVID-19 vaccines was lower (85%) compared to adults (95%). Similarly, adolescents and young people who had a COVID-19 infection had a lower vaccine utilisation (85%) than adults (95%). Likewise, in terms of being a contact of a COVID-19 case, adolescents and young people had lower COVID-19 vaccine utilisation (91%) compared to adults (97%).

Regarding environmental factors, there was significant differences in residence (*x*^2^ = 48.56, *p* = 0.00) and type of health facility (*x*^2^ = 193.61, *p* = 0.00) between adolescents and young people (15–24) and adults (25+) living with HIV as it relates to COVID-19 vaccination. Adolescents and young people residing in rural areas had higher lower vaccine utilisation (74%) compared to adults, which was unlike among adult’s urban residents (95%). In the two different age groups, getting vaccinated for COVID-19 at a central/provincial health facility had a lower utilisation proportion with 76% in adolescents and young people and 69% in adults. There was also a lower utilisation proportion among adolescents and young people (78%) accessing health services at district health facility compared to 92% for adults.

**FIGURE 2:**
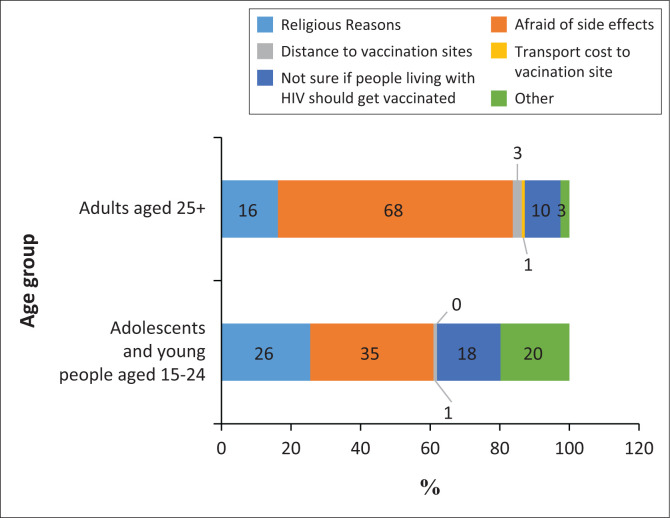
Enabling factors: Comparison of reasons of not getting vaccinated among adolescents and young people (15–24 years) and adults (25+ years) (*N* = 306).

Figure 2 shows differences in proportion of enabling factors as a reason for not getting vaccinated among adolescents and young people (15–24) and adults (25+). Regarding the enabling factors, fear of side effects was mentioned as the major reason that contributed to non-utilisation of COVID-19 vaccine among both adolescents and young people (15–24) and adults (25+). However, the proportion was higher in adults (68%) as compared to 35% in adolescents and young people. Religious reasons for non-utilisation of the COVID-19 vaccine were reported by 26% of adolescent and young people, compared to 16% in adults. Additionally, a higher proportion of adolescents and young people (18%) were unsure if PLHIV could be vaccinated for COVID-19, compared to 10% among adults.

### Multivariate analysis of differences in vaccine utilisation by age

Table 4 represents multivariate analysis of differences in vaccine utilisation by age. Regarding predisposing factors, among adolescents and young people, primary level education was significantly associated with lesser odds of COVID-19 vaccination (aOR = 0.34; 95% CI: 0.20–0.60) in comparison with secondary level education. However, this was not significant in adults (aOR = 0.93; 95% CI: 0.58–1.49). Regarding need factors, being a previous contact of a COVID-19 case was significantly associated with higher odds of COVID-19 vaccination in both age groups: among adolescents and young people (15–24 years) (aOR = 4.18; 95% CI: 1.59–10.98) and among adults (25+ years) (aOR = 2.94; 95% CI: 1.37–6.31).

**TABLE 4:** Multivariate analysis of differences in vaccine utilisation by age.

Categories	Adolescents and young people (15–24 years) living with HIV (*N* = 791)		Adults (25+) living with HIV (*N* = 1366)	
	AOR	95% CI	AOR	95% CI
**Predisposing factors**				
**Marital status**	-	-	-	-
Not married	0.79	0.48–1.31	1.16	0.77–1.74
Married	Ref	-	-	-
**Religion**	-	-	-	-
Roman Catholic	1.00	0.49–2.04	0.81	0.42–1.57
Apostolic sect	Ref	-	-	-
Pentecostal and Protestant	0.64	0.37–1.08	1.26	0.74–2.13
Other religions	1.03	0.60–1.74	1.38	0.78–2.48
**Education**	-	-	-	-
Primary	0.34	0.20–0.60***	0.93	0.58–1.49
Secondary	Ref	-	-	-
Tertiary	1.62	0.83–3.16	2.84	0.65–12.27
Did not attend school/Do not know/No answer	1.54	0.77–3.09	0.38	0.15–0.99*
**Need factors**				
**Opportunistic infection in the past 24 months**	-	-	-	-
Yes	1.47	0.54–3.99	0.96	0.47–1.95
No	Ref	-	-	-
**COVID-19 infection in the past 24 months**	-	-	-	-
Yes	0.58	0.23–1.47	1.11	0.53–2.31
No	Ref	-	-	-
**Contact of a COVID-19 case in the past 24 months**	-	-	-	-
Yes	4.18	1.59–10.98**	2.94	1.37–6.31**
No	Ref	-	-	
**Environmental factors**				
**Residence**	-	-	-	-
Rural	0.71	0.46–1.09	3.32	2.16–5.10***
Urban	Ref	-	-	-
**Type of health facility where HIV services are being accessed**	-	-	-	-
Central/provincial health facility	Ref	-	-	-
District health facility	0.82	0.36–1.81	4.74	1.43–15.66*
Primary healthcare facility	1.09	0.55–2.17	4.55	1.42–14.52*

AOR, adjusted odds ratio, CI, confidence interval, Ref, reference category.

*** , *p* < 0.001;

** , *p* < 0.01;

* , *p* < 0.05.

Regarding environmental factors, adults residing in a rural area were associated with higher odds of COVID-19 vaccination among PLHIV (aOR = 3.32; 95% CI: 2.16–5.10) in comparison with their urban counterparts. However, there was no significant association among adolescents and young people (aOR = 0.71; 95% CI: 0.46–1.09).

Accessing health services at district health facilities was significantly associated with higher odds of COVID-19 vaccination among adults living with HIV (aOR = 4.74; 95% CI: 1.43–15.66) compared to those accessing vaccination in central and provincial hospitals. However, there was no significant association among adolescents and young people (aOR = 0.82; 95% CI: 0.36–1.81).

Accessing health services at primary health facilities was significantly associated with higher odds of COVID-19 vaccination among PLHIV (aOR = 4.55; 95% CI: 1.42–14.52) than those accessing in central and provincial hospitals. However, there was no significant association among adolescents and young people (aOR = 1.09; 95% CI: 0.55–2.17).

## Discussion

The study was conducted to investigate the predisposition of people on ART to utilise the COVID-19 vaccine and factors that influence their decision. Additionally, it aimed to assess the differences in vaccine utilisation and predictors of vaccination between adolescents and young people (15–24 years) on ART and adults (25 years and older) on ART. The study found that vaccine coverage among PLHIV was 86%. A higher proportion of people who were not yet vaccinated for COVID-19 was among adolescents and young people, which was 24% (189) in comparison to adults which was 9% (117). The fear of side effects was considered as the most common reason for not getting vaccinated. Multivariate analysis revealed that residence, type of health facility and previous contact with a COVID-19 case were significantly associated in getting vaccinated among adults (25+ years) but not among the adolescents and young people (15–24 years).

The study found that the fear of side effects contributed to be the major reason for not getting vaccinated. This was however in contrast with a study in Nigeria, where vaccine acceptance among PLHIV was lower on people who were not concerned about side effects.^6^ However this was consistent with other studies which found fear of ART and COVID-19 vaccine interactions responsible for vaccine hesitancy. This was also consistent with studies among PLHIV in middle east and North Africa, in which concerns related to side effects, and potential interactions between ART were raised.^7,8^

This study contributes to the limited knowledge in literature about COVID-19 vaccination among PLHIV specifically as it relates disparities among adolescents and young people (15–24) and adults (25+). Despite a relatively high vaccine coverage of 87%. The study found a lower proportion utilisation of COVID-19 among adolescents and young people, which was 24% (189) in comparison to adults which was 9% (117). A few studies have also found younger adults COVID-19 vaccine acceptance being low.^9,10,11^ In-depth interviews showed that this was noticed as a result of the COVID-19 messaging, which targeted older age bands because COVID-19 was more severe in older age bands. This calls for the need to deliberately target the younger age bands living with HIV with vaccine information so as to improve uptake across or sub populations. In-depth interviews consistently brought that adolescents rely on caregivers for decision making such as COVID-19 vaccination. We recommend the targeting of families, communities, healthcare workers in ensuring adolescents and youth friendly information and support for caregivers for information and support for vaccinations. Adolescents and young people face all the existing challenges in accessing adolescent friendly health services. Initially, vaccination was not pushed as much for younger populations initially. So, we might need to broaden to speak to the role of families, communities, healthcare workers ensuring adolescent and youth friendly information and support for vaccinations.

In terms of limitations, it is important to notice that participants in this study do not reflect the entire population of PLHIV. The study’s main strength was that it used a relatively representative of more 2000 PLHIV. The effect of vaccination starting for adults have interfered with the uptake among adolescents. However, the authors appreciate at the time of interviewing the space had been opened up to include school children 12+ years and this includes the 15–17 who may have been side lined initially.

## Conclusion

We found that age, being a previous contact of a COVID-19 case and residence were significantly associated with COVID-19 vaccination among PLHIV. This study reports overall COVID-19 vaccine utilisation of 86% among study participants living with HIV. The study found a higher proportion of non-utilisation of COVID-19 vaccine among adolescents and young people. Factors for vaccine utilisation that were statistically significant were urban-rural residence, type of health facility and previous contact of a COVID-19 case. Continued deliberate strategies are still needed to guarantee nearly 100% or full vaccination coverage among adolescents and young living with HIV who have relative low coverage compared to adults.
